# Characterizing Repeats in Two Whole-Genome Amplification Methods in the Reniform Nematode Genome

**DOI:** 10.1155/2021/5532885

**Published:** 2021-03-06

**Authors:** S. T. Nyaku, V. R. Sripathi, K. Lawrence, G. Sharma

**Affiliations:** ^1^Department of Crop Science, College of Basic and Applied Sciences, University of Ghana, Legon, P.O. Box LG44, Ghana; ^2^Department of Biological and Environmental Sciences, Alabama A & M University, Normal AL 35762, USA; ^3^Department of Entomology and Plant Pathology, Auburn University, Auburn, AL 36849, USA

## Abstract

One of the major problems in the U.S. and global cotton production is the damage caused by the reniform nematode, *Rotylenchulus reniformis*. Amplification of DNA from single nematodes for further molecular analysis can be challenging sometimes. In this research, two whole-genome amplification (WGA) methods were evaluated for their efficiencies in DNA amplification from a single reniform nematode. The WGA was carried out using both REPLI-g Mini and Midi kits, and the GenomePlex single cell whole-genome amplification kit. Sequence analysis produced 4 Mb and 12 Mb of genomic sequences for the reniform nematode using REPLI-g and SIGMA libraries. These sequences were assembled into 28,784 and 24,508 contigs, respectively, for REPLI-g and SIGMA libraries. The highest repeats in both libraries were of low complexity, and the lowest for the REPLI-g library were for satellites and for the SIGMA library, RTE/BOV-B. The same kind of repeats were observed for both libraries; however, the SIGMA library had four other repeat elements (Penelope (long interspersed nucleotide element (LINE)), RTE/BOV-B (LINE), PiggyBac, and Mirage/P-element/Transib), which were not seen in the REPLI-g library. DNA transposons were also found in both libraries. Both reniform nematode 18S rRNA variants (RN_VAR1 and RN_VAR2) could easily be identified in both libraries. This research has therefore demonstrated the ability of using both WGA methods, in amplification of gDNA isolated from single reniform nematodes.

## 1. Introduction

The reniform nematode is most commonly found within the southern parts of U.S. and is also in many tropical and subtropical regions of the world [1]. Areas within the U.S. where this nematode has established include Alabama, Florida, Arkansas, Hawaii, Louisiana, Georgia, Mississippi, South Carolina, North Carolina, and Texas. This nematode has surpassed the root-knot nematode (*Meloidogyne incognita*) in Alabama, Louisiana, and Mississippi as the leading pest of upland cotton [2].

Development of whole-genome amplification (WGA) techniques for amplification of DNA from nanogram quantities has aided the success of most genetic research which once needed microgram quantities. These methods prove very useful in applications such as forensic science, embryonic disease diagnosis, microbial diversity, bioterrorism genome detection, and genotyping [3] and especially in sequencing genomes of single nematodes. Two of the most used WGA techniques include multiple-displacement amplification (MDA) and Omniplex methods. The MDA method utilizes *φ*29 DNA polymerase, which is highly processive, and the activities of random exonuclease-resistant primers, in the amplification reaction [4]. In the Omniplex technique, the genomic DNA is fragmented into smaller fragments (e.g., 200-2000 bases) for the generation of a library, which is then amplified. A high-fidelity polymerase is involved in the reaction which enables the library to be amplified several thousand folds. Whole-genome sequencing has been successfully used in sequencing of two human parasitic nematodes, including *Wuchereria bancrofti* [5] and *Strongyloides stercoralis* [6]. The usefulness of whole-genome amplification methods has been applied in genome sequencing projects for the reniform nematode [7, 8]. Existence of low-complexity regions (LCRs) [9] and their repeats (2.2%) within the genome of reniform nematode serves as an indication of higher levels of variation [8].

The objectives of this study were to characterize repeats in the reniform nematode genome through massively parallel genome sequencing, when two different whole-genome amplification (WGA) methods were employed.

## 2. Materials and Methods

Eggs of the reniform nematode were extracted from the roots of MicroTom tomato plants and were sterilized by immersion in 10% Clorox solution and then shaken continually for 4 minutes in a beaker. The solution was then poured through a 325-mesh sieve, nested on a 500-mesh sieve. The eggs were rinsed thrice with distilled water into a 10 ml beaker, to remove the residues of Clorox solution.

A 325-mesh sieve and a 500-mesh sieve were sterilized by autoclaving using the dry cycle (120°C for 1 hr). Extracted reniform eggs were then poured onto the 325-mesh sieve nested on the 500-mesh sieve. Clorox solutions of concentrations 0.5%, 5%, and 10% were used for sterilizing the eggs. The eggs on the 500-mesh sieve were rinsed immediately with about 300 ml of sterilized distilled water to wash off the Clorox solution for about 5 minutes and then transferred into sterilized beakers with about 10 ml of sterilized distilled water. One ml of the solution containing the sterilized eggs was placed onto the agar plates. These plates were sealed with parafilm along its edges, covered with aluminum foil, and then kept at room temperature for 2 to 4 days for the eggs to hatch. Some of the plates were also placed in the incubator set at 25°C for hatching of the eggs.

Extraction of DNA was initially from fourteen (14) single sterilized female reniform nematodes, undertaken using the DNeasy blood and tissue kit (Qiagen, Maryland, USA) according to the manufacturer's recommended protocol. Two (2.0) *μ*l of extracted DNA (~1 ng/*μ*l) was transferred into PCR tubes containing 2.5 *μ*l 10x buffer (Promega, Madison, WI, USA), 2.0 *μ*l MgCl_2_ (25 mM) (Promega, Madison, WI, USA), 0.5 *μ*l dNTPs (10 mM), 0.5 *μ*l of primer (10 *μ*m) (synthesized by MWG-Biotech AG, USA), and 0.2 *μ*l of Taq DNA polymerase (Promega, Madison, WI, USA), and the required amount of double distilled water was added to make up the final volume to 25 *μ*l. Primers 18SF (5′GCTTGTCTCAAAGATTAAGCC-3′) and 18SR (5′-TGATCCWKCYGCAGGTTCAC-3′) which amplified the 5′ one-third of the 18S rRNA gene used in the amplifications. Polymerase chain reaction (PCR) was performed in a Peltier Thermal Cycler (PTC) tetrad 2 DNA engine (Bio-Rad, Hercules, CA, USA). Polymerase chain reaction conditions were as follows: 95°C for 5 min, then 30 cycles of the following: 95°C for 30 sec, 57°C for 30 sec, and 72°C for 45 sec. The final extension phase was 72°C for 5 min. The quality of PCR products was checked by gel electrophoresis of 6 *μ*l of PCR reaction on 1% agarose gel with ethidium bromide staining. The bands were visualized and photographed under ultraviolet light. The size of each PCR product was determined by comparing it to a 100 bp DNA marker.

In order to verify the absence of bacterial contamination, DNA isolated from the nematodes was amplified using the universal bacterial primers to amplify the 16S rDNA region. Extracted DNA (2 *μ*l; ~1 ng/*μ*l) was transferred into PCR tubes containing 2.5 *μ*l 10x buffer (Promega, Madison, WI, USA.), 2.0 *μ*l MgCl_2_ (25 mM) (Promega, Madison, WI, USA.), 0.5 *μ*l dNTPs (10 mM), 0.5 *μ*l of primer (10 *μ*m) (synthesized by MWG-Biotech AG, USA), and 0.2 *μ*l of Taq DNA polymerase (Promega, Madison, WI, USA.), and double distilled water added to a final volume of 25 *μ*l. Primers PRBA338F (5′ AC TCC TAC GGG AGG CAG CAG 3′) (Lane, 1991) and PRUN518R (5′ ATT ACC GCG GCT GCT GG 3′) [10] were used in the amplifications. The PRBA338F and PRUN518R primers amplify the 338 to 518 rDNA region and contain one variable loop of rRNA. A positive control (*Pseudomonas* DNA) and a negative control (DNase-free water) were included in the amplifications. PCR reactions were performed in a Peltier Thermal Cycler (PTC) tetrad 2 DNA engine (Bio-Rad, Hercules, CA, USA). PCR conditions were as follows: 94°C for 9 min, then touched down using 9 cycles from 62°C for 30 sec to 57°C for 30 sec and 29 cycles of the following: 94°C for 30 sec, 57°C for 30 sec, and 72°C for 30 sec. The final extension phase was 72°C for 7 min. The quality of PCR products was checked by electrophoresis of 6 *μ*l of PCR reaction in 1% agarose gel with ethidium bromide staining. The gel was visualized and photographed under ultraviolet light, and the size of each PCR product was determined by comparing it with a 100 bp DNA marker.

PCR products from seven individual female nematodes were cloned into a plasmid vector using TOPO TA Cloning Kit (Invitrogen-Life Technologies, Carlsbad, CA). The ligation reaction was made up of 3 *μ*l of PCR product, 1 *μ*l of salt solution (1.2 M NaCl and 0.06 M MgCl_2_), 1 *μ*l of sterile water, and 1 *μ*l of TOPO vector. Several clones were picked for verification of inserts from PCR amplifications conducted, using each nematode clonal DNA with M13 forward and reverse primers. PCR conditions were as follows: 94°C for 5 min, then 40 cycles of the following: 94°C for 30 sec, 55°C for 1 min, and 72°C for 1 min. The final extension phase was 72°C for 10 min. Individual colonies were picked and placed in separate 1.5 ml centrifuge tubes with 1 ml of liquid LB media containing 100 *μ*g/ml of ampicillin. These were shaken at 37°C for 24 hours at 300 rpm in an Innova 4300 rotary incubator shaker (New Brunswick Scientific, Edison, NJ). Tubes containing the bacterial cells were centrifuged for 30 seconds at 13,000 rpm in a Hermle MR-2 (National Labnet Company, Woodbridge, NJ) tabletop centrifuge to obtain a cell pellet. Plasmid DNA was isolated using a QIAprep Miniprep kit (QIAGEN, Maryland, USA).

Plasmid inserts from at least ten colonies originating from each individual nematode were sequenced in both directions with two vector primers, M13 F and M13R, using the Applied Biosystems (ABI) PRISM BigDye Terminator cycle sequencing ready reaction kit (Applied Biosystems, Foster City, CA) in an ABI 3100 nucleotide sequencer in the Center for Molecular Biology at Alabama A & M University. The sequences were then screened for homology, to reniform nematode sequences using the standard nucleotide-nucleotide BLAST (blastn) on the NCBI website (http://www.ncbi.nlm.nih.gov). Sequences from nematodes having high homology to reniform nematode sequences in the GenBank were identified, and the DNA from these nematodes used for whole-genome amplification (WGA), following the manufacturer's protocol.

The DNA of four female reniform nematodes was used for WGA after confirmation of the absence of bacterial contamination. The WGA was carried out using both REPLI-g Mini and Midi kits (Qiagen, Maryland, USA), as well as the GenomePlex single cell whole-genome amplification (WGA4) kit (Sigma-Aldrich, MO, USA). Procedures for amplifications were followed according to the manufacturer's protocol. Concentrations of amplicons were determined using a TKO 100 fluorometer (Hoefer Scientific Instruments, San Francisco). The PCR products obtained from both WGA methods were further amplified using 18S and bacterial primers. Real-time PCR was then used to validate results obtained from WGA amplifications using 16S rRNA gene-specific primers in the Roche LightCycler 480 instrument (Indianapolis, IN, USA) to check for any bacterial contamination. Whole-genomic-amplified DNA was purified using the GenElute PCR clean-up kit (Sigma-Aldrich, MO, USA) and then quantified using a TKO 100 fluorometer (Hoefer Scientific Instruments, San Francisco).

Purified gDNA libraries (10 *μ*g/*μ*l) from pooled DNA of selected four single female reniform nematodes were used in 454 high-throughput sequencing at the Advanced Centre for Genome Technology (ACGT), University of Oklahoma (Norman, OK).

Gene Ontology (GO) distributions for the reniform nematode genomic sequences were determined using Blast2GO (http://www.blast2go.com) a functional annotation and visualization tool.

## 3. Results

**Figure 1:**
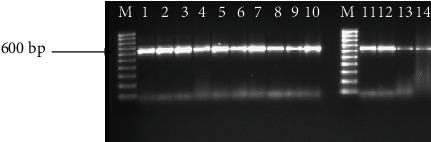
Amplification of 14 female reniform nematodes using 18S rRNA primers showing a 600 bp band. M: 100 bp molecular marker.

**Figure 2:**
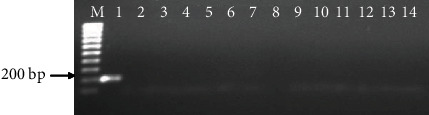
Amplification of 14 female reniform nematodes using bacterial primers. M: 100 bp marker; 1: *Pseudomonas* DNA (+ve control); 2: sterilized DNAse-free water (-ve control); 3-14 are reniform nematode amplifications.

Amplification using 18S rRNA primers produced a 600 bp band for all the 14 female reniform nematodes (Figure 1). The positive control (*Pseudomonas* DNA) amplified a 200 bp band; however, all the reniform nematode samples including the negative control (DNase-free water) showed no amplifications (Figure 2).

**Figure 3:**
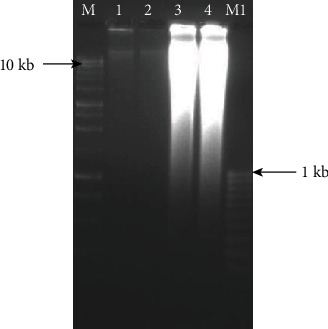
Whole-genome amplification (WGA) of 4 female reniform nematodes using REPLI-g Mini and Midi kits. M: 1 kb marker; M1: 00 bp marker; 1: 2.5 *μ*l (26 ng/*μ*l) of DNA using the Mini kit; 2: 5.0 *μ*l (26 ng/*μ*l) of DNA using the Mini kit; 3: 2.5 *μ*l (560 ng/*μ*l) of DNA using the Midi kit; 4: 5.0 *μ*l (775 ng/*μ*l) of DNA using the Midi kit).

**Figure 4:**
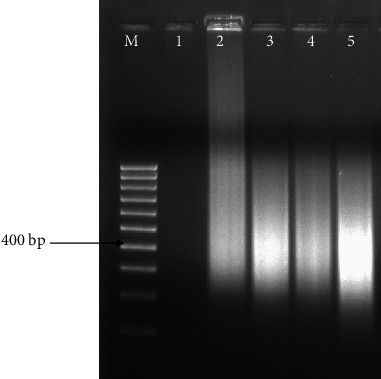
Whole-genome amplification (WGA) of 4 female reniform nematodes using SIGMA kit. M: 100 bp marker; 1: -ve control (no DNA); 2: +ve control (human genomic DNA_D7192)-45 ng/*μ*l; 3: RN DNA sample 1A (143 ng/*μ*l), 4: RN DNA sample 12A (145 ng/*μ*l); 5: RN DNA sample 13A (134 ng/*μ*l).

Whole-genome amplification (WGA) performed on four selected female nematodes, using the REPLI-g and SIGMA kits, showed distinct amplifications (Figures 3 and 4).

Real-time PCR analysis confirmed *Pseudomonas* DNA (positive control) as of a bacterial origin with the highest concentration, but no evidence of bacterial contamination was detected from the reniform nematode DNA (data not shown).

A total of 4 Mb and 12 Mb of sequence data generated from the REPLI-g and SIGMA genomic libraries were constructed from the reniform nematodes (RN). In order to reduce data redundancy, the sequences were assembled for quality and length improvements, generating 28,784 and 24,508 contigs for the REPLI-g and SIGMA libraries, respectively, using the Lasergene software (Table 1).

Repeat masker (http://www.repeatmasker.org/cgi-bin/WEBRepeatMasker) was used in characterization of the reniform nematode genome from each of the libraries and to study its organization (Tables 2 and 3), for REPLI-g and SIGMA libraries, respectively. The highest and lowest numbers of repeats within the genome of RN from the REPLI-g library were low-complexity repeats (87%) occupying a region of 130,763 bp and satellites (<1%), occupying a region of 320 bp. Simple repeats were the second highest (6%) within the reniform nematode genome occupying a 10,584 bp region. However, the reniform nematode SIGMA genomic library had retrotransposable (RTE)/Bovine-B (Bov-B) repeats being the lowest in the genome (<1%) and occupying a region of 66 bp. The highest repeats from this library were the same as those from the REPLI-g library; these were low-complexity repeats (86%), occupying a region of 304,237 bp, and the next highest repeats were simple repeats (7.2%); these occupied a 32,111 bp region.

A comparison of the genome characteristics using the repeat masker showed that the SIGMA library had four other repeat elements (Penelope (long interspersed nucleotide elements (LINE)), RTE/BOV-B PiggyBac, and Mirage/P-element/Transib); these were absent in the library generated using REPLI-g. The reniform nematode 18S rRNA variants RN_VAR1 and RN_VAR2 [11] were used in blastn analysis against the assembled reniform nematode 454 genome sequences (Table 4). A combined total of 19 hits were noted in both variants to the 454 genome sequences, and 5 contigs from the genomic sequences fully overlapped for the coverage of the full 18S rRNA gene.

**Figure 5:**
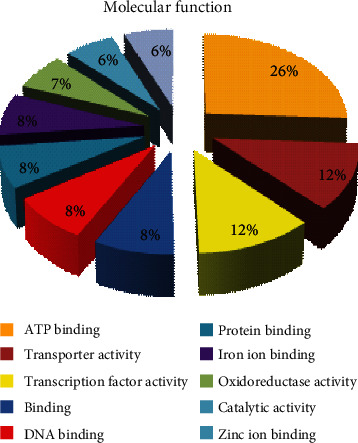
Gene Ontology (GO) distributions for reniform nematode genomic sequences as determined by Blast2GO and grouped by molecular function for the SIGMA library.

**Figure 6:**
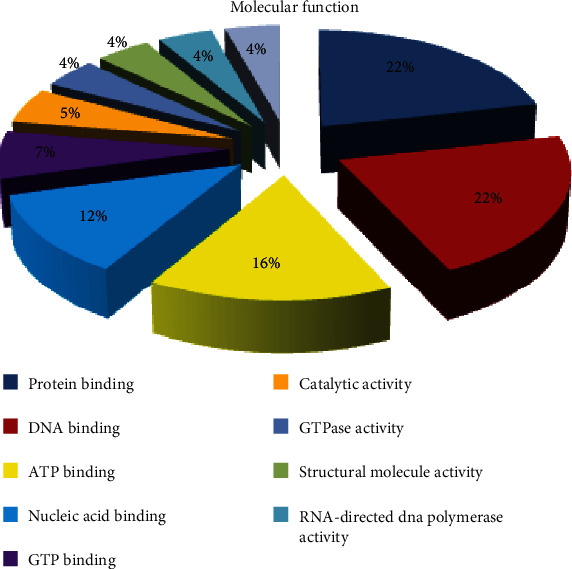
Gene Ontology (GO) distributions for reniform nematode genomic sequences as determined by Blast2GO and grouped by molecular function for the REPLI-g library.

Gene Ontology (GO) analysis performed on both genomic libraries were grouped by molecular function, biological process, and cellular component. By molecular function, ten major GO distributions were assigned with a minimum of 200 sequences with an assigned gene function for the SIGMA library. These were protein binding, ATP binding, transporter activity, transcription factor activity, binding, DNA binding, protein binding, iron ion binding, oxidoreductase activity, catalytic activity, and zinc ion binding with 875, 405, 400, 276, 274, 255, 255, 225, 212, and 210 sequences, respectively. The percentage contributions by each of these functions are shown (Figure 5). Similarly, ten major GO distributions were observed by molecular function for the REPLI-g library. These were protein binding, DNA binding, ATP binding, nucleic acid binding, GTP binding, catalytic activity, GTPase activity, structural molecule activity, RNA-directed DNA polymerase activity, and RNA binding. These had 25, 25, 18, 14, 8, 6, 5, 5, 5, and 5 sequences contributing to these functions. The percentage contributions by each of these functions are shown (Figure 6).

**Figure 7:**
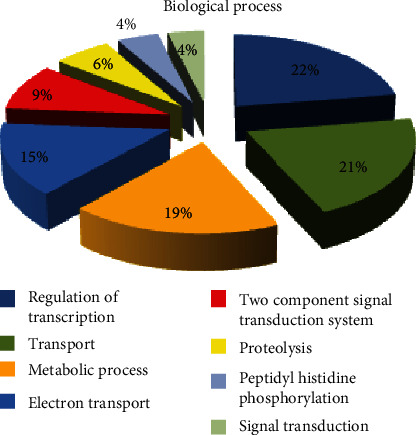
Gene Ontology (GO) distributions for reniform nematode genomic sequences as determined by Blast2GO and grouped by biological process for the SIGMA library.

**Figure 8:**
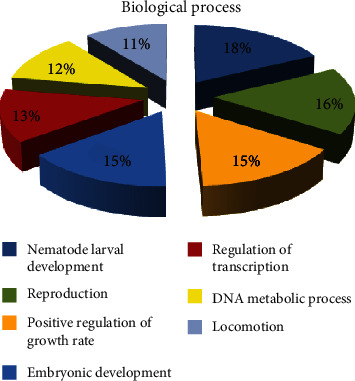
Gene Ontology (GO) distributions for reniform nematode genomic sequences as determined by Blast2GO and grouped by biological process for the REPLI-g library.

By biological process, eight major GO distributions were observed, each had a minimum of 100 sequences, contributing to that function or annotation for the SIGMA library. These include regulation of transcription, transport, metabolic process, electron transport, two-component signal transduction system, proteolysis, peptidyl-histidine phosphorylation, and signal transduction with 637, 585, 525, 412, 262, 175, 125, and 110 sequences, respectively. The percentage contributions by each of these processes are shown (Figure 7). However, by biological process, seven major GO distributions were rather observed for the REPLI-g library, each of which had a minimum of 9 sequences contributing to that function or annotation. These were nematode larval development, reproduction, positive regulation of growth, embryonic development, regulation of transcription, DNA metabolic process, and locomotion with 15, 14, 13, 13, 11, 10, and 9 sequences, respectively. The percentage contributions by each of these processes are shown (Figure 8).

**Figure 9:**
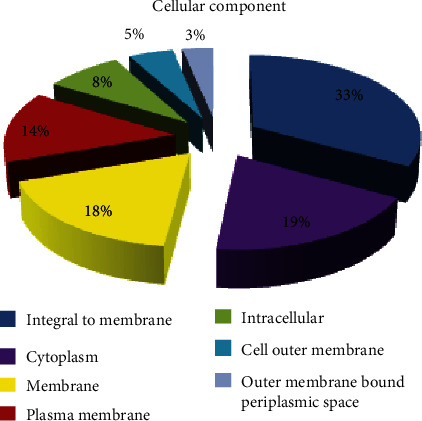
Gene Ontology (GO) distributions for reniform nematode genomic sequences as determined by Blast2GO and grouped by cellular component for the SIGMA library.

**Figure 10:**
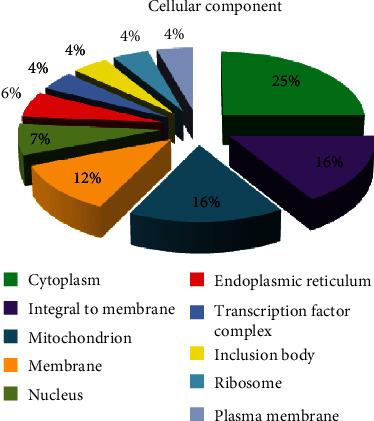
Gene Ontology (GO) distributions for reniform nematode genomic sequences as determined by Blast2GO and grouped by cellular component for the REPLI-g library.

By cellular component, seven major GO distributions were observed for the SIGMA library, each of which had a minimum of 100 sequences contributing to that function or annotation. These were integral to membrane, cytoplasm, membrane, plasma membrane, intracellular membrane, cell outer membrane, and outer membrane-bound periplasmic space with 1,000, 575, 540, 425, 250, 145, and 100 sequences, respectively. The percentage contributions by each of these components are also presented for detailed examination (Figure 9). The REPLI-g library however had ten major GO distributions for the cellular component, each of which had a minimum of 3 sequences contributing to that function or annotation. Their organellar and membrane-bound distributions were cytoplasm, integral to membrane, mitochondrion, membrane, nucleus, endoplasmic reticulum, transcription factor complex, inclusion body, ribosome, and plasma membrane with 17, 11, 11, 8, 5, 4, 3, 3, 3, and 3 sequences, respectively. The percentage contributions by each of these components are also presented (Figure 10).

## 4. Discussion

Mobile genetic elements (retrotransposons) have the ability of generating DNA through reverse transcription of RNA; this DNA is then inserted into the eukaryotic genome. The horizontal gene transfer between bird and nematode genomes took place in two pantropical waves, 425–22 million years ago (Myr ago) involving the Brugia/Wuchereria lineage and 420–17 Myr ago involving the Loa lineage [12]. A number of these elements were observed within both genomic libraries of the reniform nematode. Classes of retrotransposons include long-terminal-repeat (LTR) retrotransposons; these elements have mechanisms possessed by vertebrate retroviruses. Elements BEL/Pao and Gypsy/DIRS1 were present in both genomic libraries of the reniform nematode. Depending on the type of reverse transcriptase (RT) domain possessed by the LTR retrotransposons, these were categorized into Ty1/copia, Bel, and Ty3/gypsy groups. In *Saccharomyces cerevisiae*, Ty1 and Ty3 are very well studied and characterized; on the other hand, copia, Bel, and Gypsy are found in *Drosophila melanogaster*.

Another group is the tyrosine recombinase retrotransposons which have similar properties to those of LTR retrotransposons; however, they possess integrase instead of recombinase, as they integrate into the host chromosomes. The second group is the non-LTR retrotransposons, which do not possess either inverted or tandem terminal repeats; these have poly(A) tails at their 3′ ends, and their 5′ ends have variable deletions (5′ truncations). These elements encode open reading frames (ORFs), which are prone to mutations [13]. Examples of these elements are LINEs (long interspersed nucleotide elements), which are autonomous and about 5-10 kb in length; the other is SINEs (short interspersed nucleotide elements), which are nonautonomous and about 100-400 bp in length [14]. The Penelope-like retrotransposons and LINEs found in *Drosophila virilis* [15]. This element inserts into the host genome and their structures that are very diverse compared to other classes of retrotransposons. These elements have the Uri domain (GIY-YIG), and for reverse transcription, they make use of the ends of chromosomes to serve as primers. Two Penelope-like retrotransposons were identified in only the SIGMA genomic library of the reniform nematode. The third group of transposable elements is DNA transposons; these have short terminal inverted repeats (TIRs) of about 2-10 bp at their terminals, with a long ORF, which codes for protein domains possessing transposase (TR) and DNA binding mechanisms. These elements were present in both genomic libraries of the reniform nematode. Transposable elements could either be autonomous (1000-4000 bp) or nonautonomous (100-3000 bp); i.e., an example is Miniature Inverted Repeat Transposable elements (MITEs). Transposition of DNA transposons occurs in a variety of ways; however, their duplications occur when DNA is being replicated [16]. The “cut-and-paste” mode of action is usually utilized during DNA transposition in majority of eukaryotes; here, the element is cleaved and transported to another location by the TR. However, the rolling circle (RC) mechanism is used by other eukaryotic elements during DNA transposition; this is similar to that occurring in prokaryotes [17]. In a recent study, DNA transposons or LTR retroelements (20.6 Mb and 11.3 Mb, respectively) were identified in reniform nematode genomic assembly (RREN1.0, GCA_001026735.1) of size 314 Mb [8].

The majority of repeats within both reniform nematode genomic libraries were low-complexity repeats (>86%). Low-complexity regions (LCRs) occur in many genomes and among protein families [9]. The diversity occurring among the amino acid sequences of these regions is low, and any variation occurring may range from areas with one or many amino acids at specific positions [18]. Meiotic recombination has been implicated in the fast evolutions of LCRs [19]. Repeats are also present in genomes of prokaryotes, contributing to genetic variations in these organisms [20]. The highest source of variation in LCRs is from the tandem repeats which could expand and contract [21]. The prevalence of high low-complexity repeats within the reniform nematode genome suggests high levels of variation in its genome. These low-complexity repeats occupied about 2.2% of the reniform genome [8].

Simple repeats were the next highest repeats within the reniform nematode genome; these were about 6% and 7.2% of total repeats for the REPLI-g and SIGMA libraries. These simple repeats could be either be microsatellites or minisatellites. Microsatellites contribute to evolution within organisms [22] and are also subjected to changes in their lengths (replication slippage). Replication slippage does not occur frequently; its occurrence is once every 1,000 generations [23]. The presence of simple repeats in the reniform nematode genome may serve as markers for the identification of resistance in plants attacked by this nematode. Another transposon identified in the SIGMA library is the PiggyBac transposon. This has been used in transposon mutagenesis in insects (e.g., *Drosophila melanogaster*). The Piggyback transposase activity was observed in *D. melanogaster* when the mutator elements in this organism present on the X chromosome in males were used in providing this activity through a *Hermes*-based jump starter element; the *α*-*1-tubulin* promoter was used in this process [24]. In transposon mutagenesis, transposal elements are able to insert themselves into genomic loci resulting in gene disruption.

## 5. Conclusion

This research has revealed the ability of using both WGA methods in amplification of gDNA from individual reniform nematodes with both techniques useful in increasing DNA concentrations and identification of repeats, which can also be applied in other species of nematodes. The highest repeats in both libraries were low complexity; however, the lowest repeats for the REPLI-g and SIGMA libraries were satellites and RTE/BOV-B repeats, respectively.

## Figures and Tables

**Table 1:** Reniform nematode assembly characteristics for REPLI-g and SIGMA genomic libraries.

Assembly characteristics	REPLI-g	SIGMA
Total number of contigs	28,784	24,508
Assembly combined length	4,418,499 bp	12,732,631 bp
Number of sequences < 200 bp	21,125	1903
Number of sequences between 200 bp & 1 kb	7656	20,305
Number of sequences between 1 kb & 5 kb	3	2243
Number of sequences between 5 kb & 10 kb	0	57

**Table 2:** Summary of repeats within the reniform nematode genome using the REPLI-g library.

Type of element	No. of elements	Length occupied (bp)
Retroelements	50	5682
L2/CRI/Rex (LINEs)	8	608
BEL/Pao (LTR)	6	305
Gypsy/DIRS1 (LTR)	36	4769
DNA transposons	70	5188
Hobo-activator	10	829
Tc1-IS630-Pogo	47	3615
MuDR-IS905	8	490
Unclassified	6	447
Small RNA	39	6023
Satellites	2	320
Simple repeats	231	10,584
Low complexity	3311	130,763

Total	3824	169,623

**Table 3:** Summary of repeats within the reniform nematode genome using the SIGMA genomic library.

Type of element	No. of elements	Length occupied (bp)
Retroelements	79	12,340
Penelope (LINEs)	2	227
L2/CRI/Rex (LINEs)	26	2956
RTE/BOV-B (LINEs)	1	66
BEL/Pao (LTR)	5	251
Gypsy/DIRS1 (LTR)	45	8840
DNA transposons	79	6882
Hobo-activator	13	1045
Tc1-IS630-Pogo	47	4716
MuDR-IS905	7	322
PiggyBac	2	103
Other (Mirage, P-element, Transib)	2	105
Unclassified	36	6549
Small RNA	121	24,200
Satellites	8	1785
Simple repeats	491	32,111
Low complexity	5890	304,237

Total	6854	406,735

**Table 4:** Summary of blast hits among reniform nematode 18S variants and 454 genomic sequences.

RN 18S rRNA variant	REPLI-g 454 sequences	SIGMA 454 sequences
Total number of hits (RN_VAR1)	54	244
Overlapping contigs (nonredundant) (RN_VAR1)	16	16
Total number of hits (RN_VAR2)	56	244
Overlapping contigs (nonredundant) (RN_VAR2)	16	13

## Data Availability

The raw reads for RN genomic sequences were submitted to the NCBI Sequence Read Archive (accession Nos. SRX099033 and SRX099034) for REPLI-g and SIGMA libraries, respectively.

## References

[1] Davis R. F., Koenning S. R., Kemerait R. C., Cummings T. D., Shurley W. D. (2003). Rotylenchulus reniformis management in cotton with crop rotation. *Journal of Nematology*.

[2] Robinson A. F. (2007). Reniform in U.S. cotton: when, where, why, and some remedies. *Annual Review of Phytopathology*.

[3] Hawkins T. L., Detter J. C., Richardson P. M. (2002). Whole genome amplification - applications and advances. *Current Opinion in Biotechnology*.

[4] Dean F. B., Hosono S., Fang L. (2002). Comprehensive human genome amplification using multiple displacement amplification. *Proceedings of the National Academy of Sciences*.

[5] Small S. T., Reimer L. J., Tisch D. J. (2016). Population genomics of the filarial nematode parasite *Wuchereria bancrofti* from mosquitoes. *Molecular Ecology*.

[6] Kikuchi T., Hino A., Tanaka T. (2016). Genome-wide analyses of individual *Strongyloides stercoralis* (Nematoda: Rhabditoidea) provide insights into population structure and reproductive life cycles. *PLoS Neglected Tropical Diseases*.

[7] Nyaku S. T., Sripathi V. R., Kantety R. V. (2014). Characterization of the reniform nematode genome by shotgun sequencing. *Genome*.

[8] Showmaker K. C., Sanders W. S., Eves-van den Akker S. (2019). A genomic resource for the sedentary semi-endoparasitic reniform nematode, Rotylenchulus reniformis Linford & Oliveira. *Journal of Nematology*.

[9] Coletta A., Pinney J. W., Solís D. Y., Marsh J., Pettifer S. R., Attwood T. K. (2010). Low-complexity regions within protein sequences have position-dependent roles. *BMC Systems Biology*.

[10] Muyzer G., De Waal E. C., Uitterlinden A. G. (1993). Profiling of complex microbial populations by denaturing gradient gel electrophoresis analysis of polymerase chain reaction-amplified genes coding for 16S rRNA. *Applied and Environmental Microbiology*.

[11] Nyaku S. T., Sripathi V. R., Kantety R. V., Gu Y. Q., Lawrence K., Sharma G. C. (2013). Characterization of the two intra-individual sequence variants in the 18S rRNA gene in the plant parasitic nematode, *Rotylenchulus reniformis*. *PLoS One*.

[12] Suh A. C. C., Witt J., Menger K. (2016). Ancient horizontal transfers of retrotransposons between birds and ancestors of human pathogenic nematodes. *Nature Communications*.

[13] Eickbush T. H., Jamburuthugoda V. K. (2008). The diversity of retrotransposons and the properties of their reverse transcriptases. *Virus Research*.

[14] Tang H. (2007). Genome assembly, rearrangement, and repeats. *Chemical Reviews*.

[15] Evgen'ev M. B., Zelentsova H., Shostak N. (1997). Penelope, a new family of transposable elements and its possible role in hybrid dysgenesis in *Drosophila virilis*. *Proceedings of the National Academy of Sciences*.

[16] Brookfield J. F. Y., Sherratt D. J. (1995). Transposable elements as selfish DNA. *Mobile Genetic Elements*.

[17] Kapitonov V. V., Jurka J. (2001). Rolling-circle transposons in eukaryotes. *Proceedings of the National Academy of Sciences*.

[18] DePristo M., Zilversmit M., Hartl D. (2006). On the abundance, amino acid composition, and evolutionary dynamics of low- complexity regions in proteins. *Gene*.

[19] Marcotte E. M., Pellegrini M., Ng H. L., Rice D. W., Yeates TO, Eisenberg D. (1999). Detecting protein function and protein-protein interactions from genome sequences. *Science*.

[20] Moxon E. R., Rainey P. B., Nowak M. A., Lenski R. E. (1994). Adaptive evolution of highly mutable loci in pathogenic bacteria. *Current Biology*.

[21] Fondon J., Garner H. (2004). Molecular origins of rapid and continuous morphological evolution. *Proceedings of the National Academy of Sciences*.

[22] Vinces M. D., Legendre M., Caldara M., Hagihara M., Verstrepen K. J. (2009). Unstable tandem repeats in promoters confer transcriptional evolvability. *Science*.

[23] Weber J. L., Wong C. (1993). Mutation of human short tandem repeats. *Human Molecular Genetics*.

[24] Horn C., Offen N., Nystedt S., Hacker U., Wimmer E. A. (2003). piggyBac-based insertional mutagenesis and enhancer detection as a tool for functional insect genomics. *Genetics*.

